# Digital Phenotypes for Understanding Individuals' Compliance With COVID-19 Policies and Personalized Nudges: Longitudinal Observational Study

**DOI:** 10.2196/23461

**Published:** 2021-05-27

**Authors:** Ahmed Ibrahim, Heng Zhang, Sarah Clinch, Ellen Poliakoff, Bijan Parsia, Simon Harper

**Affiliations:** 1 Department of Computer Science The University of Manchester Manchester United Kingdom

**Keywords:** behavior, compliance, COVID-19, digital phenotyping, nudges, personalization, policy, sensor, smartphone

## Abstract

**Background:**

Governments promote behavioral policies such as social distancing and phased reopening to control the spread of COVID-19. Digital phenotyping helps promote the compliance with these policies through the personalized behavioral knowledge it produces.

**Objective:**

This study investigated the value of smartphone-derived digital phenotypes in (1) analyzing individuals’ compliance with COVID-19 policies through behavioral responses and (2) suggesting ways to personalize communication through those policies.

**Methods:**

We conducted longitudinal experiments that started before the outbreak of COVID-19 and continued during the pandemic. A total of 16 participants were recruited before the pandemic, and a smartphone sensing app was installed for each of them. We then assessed individual compliance with COVID-19 policies and their impact on habitual behaviors.

**Results:**

Our results show a significant change in people’s mobility (*P*<.001) as a result of COVID-19 regulations, from an average of 10 visited places every week to approximately 2 places a week. We also discussed our results within the context of nudges used by the National Health Service in the United Kingdom to promote COVID-19 regulations.

**Conclusions:**

Our findings show that digital phenotyping has substantial value in understanding people’s behavior during a pandemic. Behavioral features extracted from digital phenotypes can facilitate the personalization of and compliance with behavioral policies. A rule-based messaging system can be implemented to deliver nudges on the basis of digital phenotyping.

## Introduction

### Background

COVID-19 is a highly contagious disease with confirmed cases in more than 188 countries as between December 2019 and June 2020, resulting in a global pandemic [1]. To control the spread of COVID-19, governments have enforced behavioral policies, such as stay-at-home and social distancing measures, which limit the usual patterns of human interaction [2,3]. The potential risk of problems with social isolation [4] complicates the implementation of these policies, which places an additional responsibility on governments to maintain mental health throughout the pandemic.

Currently, governments rely on communication campaigns to persuade people to adhere to COVID-19 behavioral policies and reduce disease spread. Health agencies, such as the National Health service (NHS) in the United Kingdom, design communication in a way that encourages the application of the promoted behaviors while avoiding problems related to social isolation. This approach to communications design employs behavioral insights derived from scientific studies to deliver behavioral guidance [5]. The communications resulting from this process are called “nudges” [6].

Despite the critical role of these campaigns in elevating community awareness, they are not designed to reflect differently when people exhibit different behavioral responses to the promoted procedures. Digital devices including smartphones can be used to recognize behavioral differences. Accordingly, communications can be personalized and contextualized on the basis of the individual’s behavior. Smartphones facilitate the capturing of behavioral features through the continuous and unobtrusive collection of sensor and interaction data; this process is known as “digital phenotyping.”

In this study, we show how an individual’s behavioral reactions to COVID-19 policies can be observed through digital phenotyping. Subsequently, we suggest a personalized way of delivering nudges designed around the individual’s reactions to the enforced regulations. We report 2 longitudinal studies that started before the outbreak of the pandemic to collect digital phenotypes. Our studies allow us to observe the impact on the overall behavior before and during the outbreak. Additionally, we observed the impact of COVID-19 on habitual behaviors and the uptake of new apps.

Our primary research contribution is the introduction of an approach that employs behavioral differences derived from digital phenotyping in the design of personalized nudges. Although we did not conduct an experiment to measure the real-time effects of personalized nudges, the proposed nudges conform to the general guidelines in behavioral science and are expected to improve individual compliance to them. Moreover, the development of mental health issues as a result of lockdown policies can be observed through digital phenotyping and better addressed through personalized nudges.

### Related Work

With the popularity and evolution of personal electronic devices, people are producing an increasing number of digital footprints such as those generated through web-based communication and mobile device usage. These footprints can be linked and analyzed with clinical data to create an individualized, nuanced view of human disease, which is called a “digital phenotype” [7]. In 2015, a digital phenotype was defined by Jukka-Pekka Onnela as the “moment-by-moment quantification of the individual-level human phenotype in-situ using data from smartphones and other personal digital devices” [8]. Digital phenotyping has become one of the most innovative approaches to enhance health and wellness via human-computer interactions through digital technology.

Nowadays, smartphones have become the one of the ideal tools for digital phenotyping. Smartphones are the hub of personal communication, and almost everyone has a smartphone. Although smartphones are not specially designed for behavioral research, they can collect a large amount of related data directly and instantly with ecological validity. Social interaction on smartphones, including calls, messages, emails, and social media usage, can be captured without difficulty. Thus, social sensing could be less intrusive on smartphones than on any other device. Embedded multiple and power sensors also empower smartphones as an efficient tool to record the surrounding social context. For example, raw data from sensors such as microphones, the global positioning system (GPS), and accelerometers can be gathered and interpreted as conversation engagement, mobility patterns, and the number of encounters to infer social interaction occurring outside of smartphones. Thus, smartphones could be one of the most applicable ways of passive societal digital phenotyping.

Digital phenotyping on smartphones has been utilized in various fields, especially psychological and health-related studies. Abdullah et al [9] collected phone usage patterns to detect and predict discrepancies in sleep rhythms. Furthermore, LiKamWa et al [10] analyzed call, message, or email contacts and location clusters from smartphones to infer users’ daily mood. Farhan et al [11] combined the locations and activities from participants’ smartphones to predict depression. Boukhechba et al [12] explored the association of social anxiety with GPS and communication patterns. To confirm the findings and observations of passively collected smartphone data, all these studies asked for participants’ input through various means including interviews, focus groups, and questionnaires. All these studies claimed to have relatively high accuracy. Albeit with different aims, our study similarly implemented these smartphone monitoring technologies. We collected data before and during the COVID-19 lockdown, which provided us an opportunity to observe individual behavioral changes. We also conducted interviews with our study participants to verify our findings.

## Methods

### Methods Overview

We used behavioral indicators for the COVID-19 policies as proxies that would help us observe the adoption of the desired change by people. Our approach relies on transforming raw smartphone data collected longitudinally (ie, digital phenotypes) into behavioral features. Distance travelled and time spent at home by a person are examples of features derived from raw location data (ie, timestamped longitude and latitude attributes). The detection of behavioral indicators is achieved at the level of behavioral features rather than the raw data. This is because behavioral indicators are manifested at a higher level of human understanding expressible by those features. In the following section, we detail the behavioral features and their roles in recognizing the behavioral indicators of the proposed policies.

For this disease, transmitted through close contact, reducing the possibility of an uninfected person having physical contact with an infected person may be the only effective way to suppress the transmission of the disease. Since the onset of the COVID-19 pandemic, governments worldwide enforced a series of behavioral policies based on this concept to control the spread of this highly infectious disease. For example, the government of the United Kingdom instructed individuals to stay home as much as possible, to limit contact with those from other households, and to maintain distance from others when stepping out of home (2 meters apart where possible) [13]. Other measures include school closures, working from home, cancellation of mass gatherings, and travel restrictions. These policies are referred to as “social distancing” or “physical distancing” policies.

### Stay-at-Home Measures

Deriving behavioral indicators of social distancing from smartphone data was our primary consideration. There are some existing studies on the mobility responses to COVID-19; for instance, a previous study [14] analyzed public geolocated Twitter data to measure the travel behaviors of users. Allcott et al [15] combined surveys and GPS foot traffic patterns to observe partisan differences in social distancing. They reported a substantial reduction in the mobility of people in the United States, albeit with partisan gaps in beliefs and behavior. Similarly, we can expect that our participants should spend almost all their time at home and to limit the time and number of places when stepping out, which is usually only for essential shopping owing to the implementation of social distancing measures. These behavioral changes can be acquired from raw GPS data. Since participants’ smartphones record latitude and longitude attributes continuously, their distance from home can always be calculated. Thus, we can determine the time and frequency of their trips outside of home.

Furthermore, social distancing measures can bring about adverse effects, especially on mental health. Some of these reported effects include stress, anxiety and depression, and panic [16]. To maintain mental well-being and while at home, people may find alternative methods of communication to replace their regular face-to-face interactions. Phone calls, messages, video chatting, and social media are possible substitutions people may choose; accordingly, a potential increase in the use of these communication methods is expected. With the various data sources, we could draw a comprehensive and personalized picture of how people react to the impact of COVID-19 restrictions.

### Social Distancing Measures

Social distancing implies that people should meet fewer people than they would during normal times. Bluetooth signals are an effective reference for face-to-face interaction recorded on smartphones. Nowadays, almost everyone carries a smartphone, and almost every smartphone is equipped with Bluetooth technology, which scans surrounding signals and reports its identity continuously in a short range. Thus, every newly captured Bluetooth entry could potentially represent a new person in close proximity [17]. This technology has been wildly used in the field to estimate face-to-face proximity [18]. Although it is not fully accurate because of the physical position of the smartphone and surrounding environments, it can still provide a trend that people have less face-to-face interactions. Hence, owing to the social distancing policy, a reduction in the number of unique Bluetooth signals is expected. Theoretically, this would indicate whether our participants adhere to the rules of staying at home and avoiding others visiting their household.

Moreover, social distancing has also affected people when they go for essential shopping. Many grocery stores have a limited number of people in their branches and have introduced directional floor markings to help shoppers maintain a 2-meter distance from one another [19]. This policy could reduce the capacity of crowded grocery stores, and fewer people are expected to be in close proximity to our participants compared to the time before social distancing measures were implemented. Thus, from Bluetooth signals, we could expect a reduction in the number of unique devices from a single scan.

### Experiments

We report results from 2 longitudinal studies conducted to gather smartphones’ digital phenotypes. Both studies were underway prior to, and continued through, large-scale transmission of COVID-19 and associated social distancing behaviors.

### Participants

The studies were reviewed and approved by the Department of Computer Science Ethics Committee at the University. A total of 16 participants were recruited (4 males and 4 females per experiment) through the university database and websites. The 2 experiments recruited individuals from different populations in the United Kingdom: (1) students and (2) patients with a diagnosis of Parkinson disease (aged 63-75 years).

The 2 studies used smartphones to capture data on the participants’ activities. Both experiments rely on the same sensing platform.

### Instrument

In this study, we used smartphones as independent sensing tools to retrieve participants’ behavioral data. The AWARE sensing platform [20] and developed plug-ins were deployed on participants’ smartphone as a monitor app. Under the approval of the ethics committee, different kinds of data, including calls, messages, social media app usage, smartphone usage, notifications, locations, Bluetooth signals, and Wi-Fi signals were collected passively. The content of sensitive communications, such as calls, messages, and conversations, was not recorded. All these data were processed to maintain the anonymity and confidentiality of all participants. All data sources are summarized in Table 1.

Participants were asked to attend an introductory interview to obtain information on our study and to clarify any of their doubts. On obtaining formal approval from the participants, the AWARE app was installed on their smartphones. Participants were asked to keep the installed app running and use their phones as they normally do. An offline analysis was conducted on data synced with the backend AWARE server.

**Table 1:** Sources of the collected digital phenotypes with data descriptions.

Source	Data
Global positioning system	Location coordinates (longitude and latitude).
Weather	Temperature, humidity, pressure, wind speed, cloudiness, amount of rain and snow, and times of sunrise and sunset.
Apps	App usage data.
Notifications	All notifications generated by any app installed on the phone.
Screen usage	Screen interactions and visited websites.
Wi-Fi signals	Access points.
Bluetooth signals	Nearby devices.
Battery status	Charging status, charging start time, charging end time, discharging start time, and discharging end time.
Calls	Call types (outgoing, incoming, and missed calls) and times. Numbers are stored in an encrypted format.
Keyboard	Time and the typed letters (numbers and emails are replaced with asterisks).
Consent form	Name and signature.
Questionnaire	We asked about interests, which we inferred from the data.
Activity recognition	Tilting, running, being within a vehicle, walking, being on a bicycle, and traveling on foot.

## Results

### Results Overview

**Figure 1:**
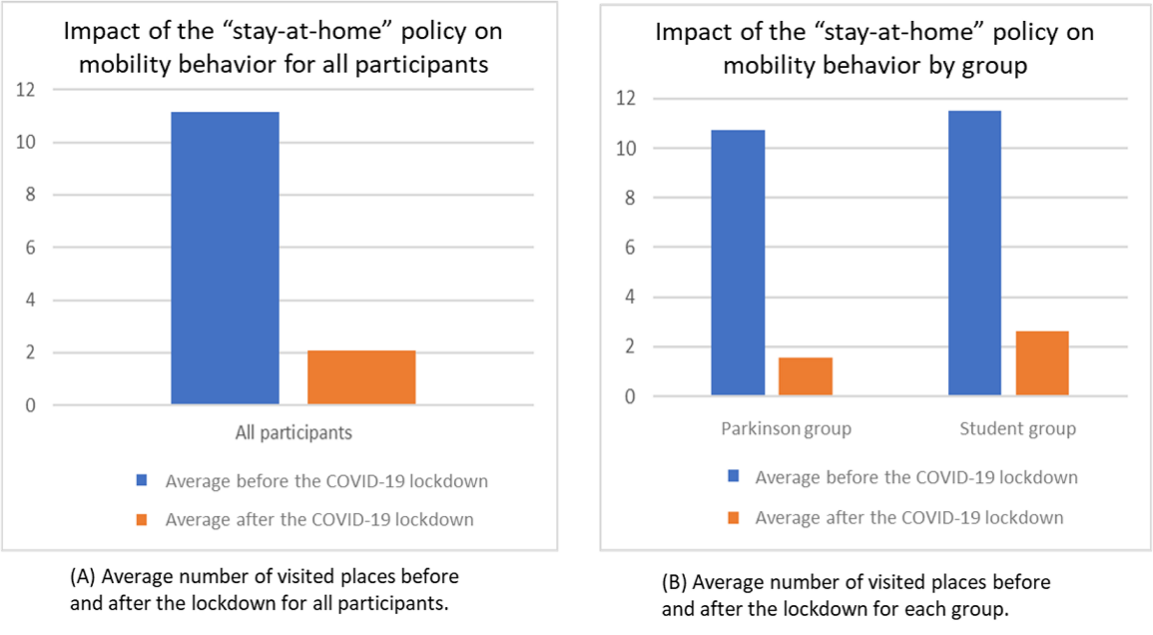
Impact of the stay-at-home policy on mobility behavior.

This section discusses the results obtained from the responses to the stay-at-home and social distancing policies. To show how digital phenotyping can help understand behavioral responses to these policies, we selected a prototypical participant who exemplified the general behavioral responses exhibited by all participants, in each subsection, except for Figure 1, which represents all participants. Our experiments started at different times; therefore, the lockdown timelines for each participant may differ. The behavioral responses to COVID-19 were captured despite the differences in the lockdown week. It was intended per our experimental design to have participants adhere to these policies at different times because participants were individually assessed, and no extrapolation among other participants was intended.

### Stay-at-Home Measures

Mobility patterns for participants in both experiments significantly decreased as a result of the compliance with the stay-at-home policy (*P*<.001) (Figure 1A). Before the lockdown, the average number of places visited was slightly lesser among patients with Parkinson disease than among the students (Figure 1B). However, a patient with Parkinson disease and a student may exhibit similar responses to the stay-at-home policy. Thus, individuals of the same group can exhibit a pattern that is different from the average behavior of their corresponding groups. Thus, individual analysis of digital phenotypes would help better understand people’s compliance with the suggested policies.

Participants exhibited similar behavioral responses to COVID-19 regulations. We selected a participant who exemplifies the behavioral responses to present the results. We divided the participant’s behavior window by week (Monday to Sunday), such that a whole cycle of a weekly social routine could be acquired. The stop point detection algorithms were applied for raw GPS data, such that the place of residence of the participant could be extracted. We used the algorithm proposed by Li et al [21] to extract stop points. The algorithm processes data points sequentially, and stop points are defined on the basis of predefined time and distance thresholds. Furthermore, we considered the location where participants spend most of their time of the day as their home. We used Foursquare [22] to determine the names of places, which allows for a better understanding of location semantics. By summing up the calculated results of the algorithm, the length of time participants spend at home and time spent by participants outside of home per week were obtained.

**Figure 2:**
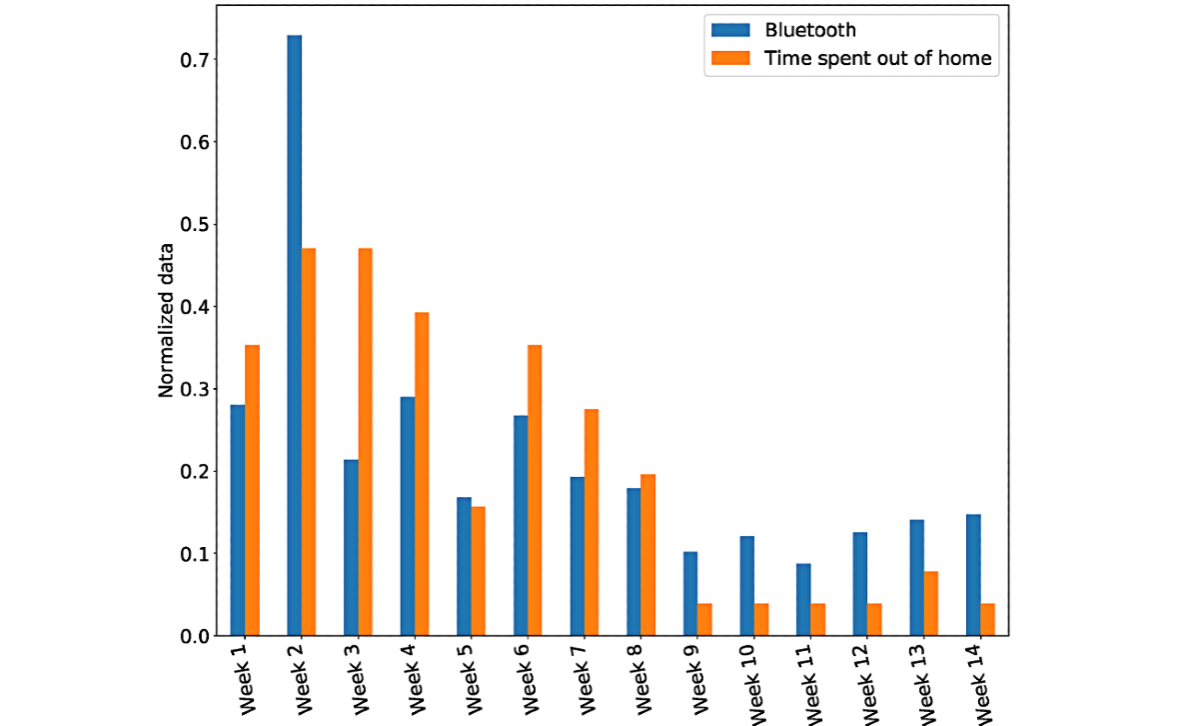
Normalized unique Bluetooth signals and time spent out of home of a participant before and after the lock-down.

Another indicator is Bluetooth signals. As mentioned before, a scanned unique Bluetooth device could represent a person in close proximity. With everyone staying at home, fewer new identified Bluetooth entries were expected to be recorded. The time spent outside of home was usually below 30 minutes, but identified Bluetooth entries were all above 1000. To easily observe the similar trend of time spent outside of home and the number of new identified Bluetooth entries, we normalized the actual data so they can be plotted on the same graph. As illustrated in Figure 2, a clear boundary was observed, in that the participant went outside of home fewer times and presented decreased unique Bluetooth entries. Although fluctuations continue, the edge appeared around week 9; that is, March 15-22. This was the week before a lockdown was officially declared in the United Kingdom. Thus, it was observed that this participant perceived the stay-at-home policy and obeyed it objectively.

**Figure 3:**
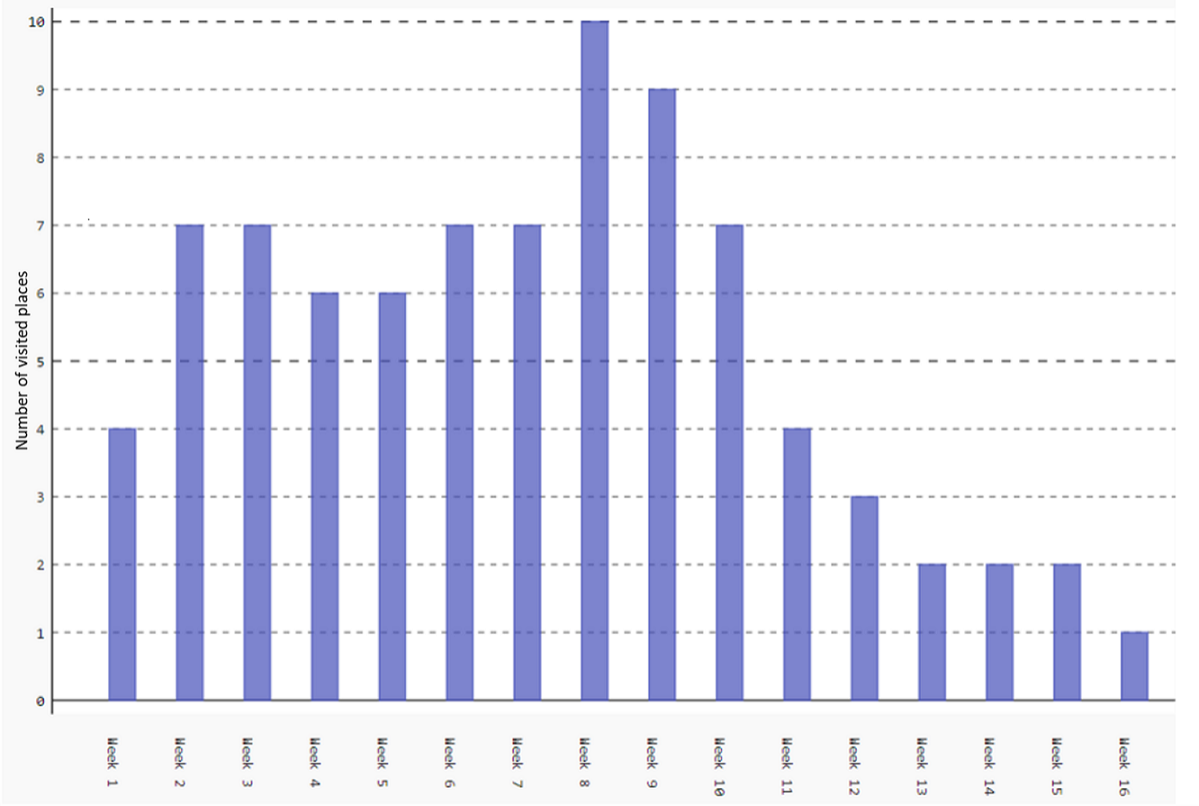
Location visited by a participant before and after the lockdown.

Figure 3 shows the impact of the “stay-at-home” policy on participant mobility. The figure represents the mobility behavior of participants who reside in the United Kingdom. Starting from week 12, the number of visited locations drastically decreased from an average of 7 locations to 2 locations. The 2 locations are the participant’s home and a grocery store. To motivate this participant to comply with the stay-at-home policy, options for the delivery of grocery items or shopping times can be communicated.

### Social Distancing Measures

**Figure 4:**
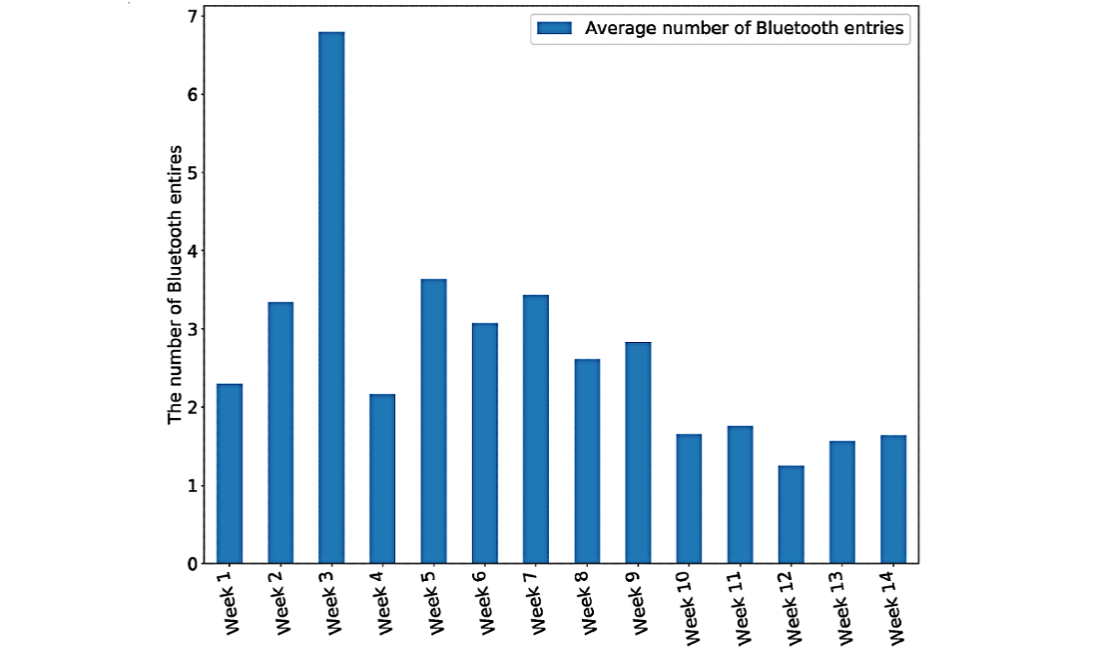
Average 1-time Bluetooth entries before and after the lockdown.

As described before, in accordance with the social distancing policy, people have to stay further away from each other than they would during normal times. Because of the capability of the Bluetooth technology, fewer scanned entries would be expected at a time. In this example, we also separated the data into natural weeks and combined all Bluetooth records within that week. Then, we divided this number by the total times for the scans to calculate the average 1-time Bluetooth discovery. As shown in Figure 4, the average 1-time Bluetooth entries decreased around week 9, which is the first week of the official lockdown in the United Kingdom. This potentially indicates that the participant maintained social distance with others and met fewer people during the lockdown.

**Figure 5:**
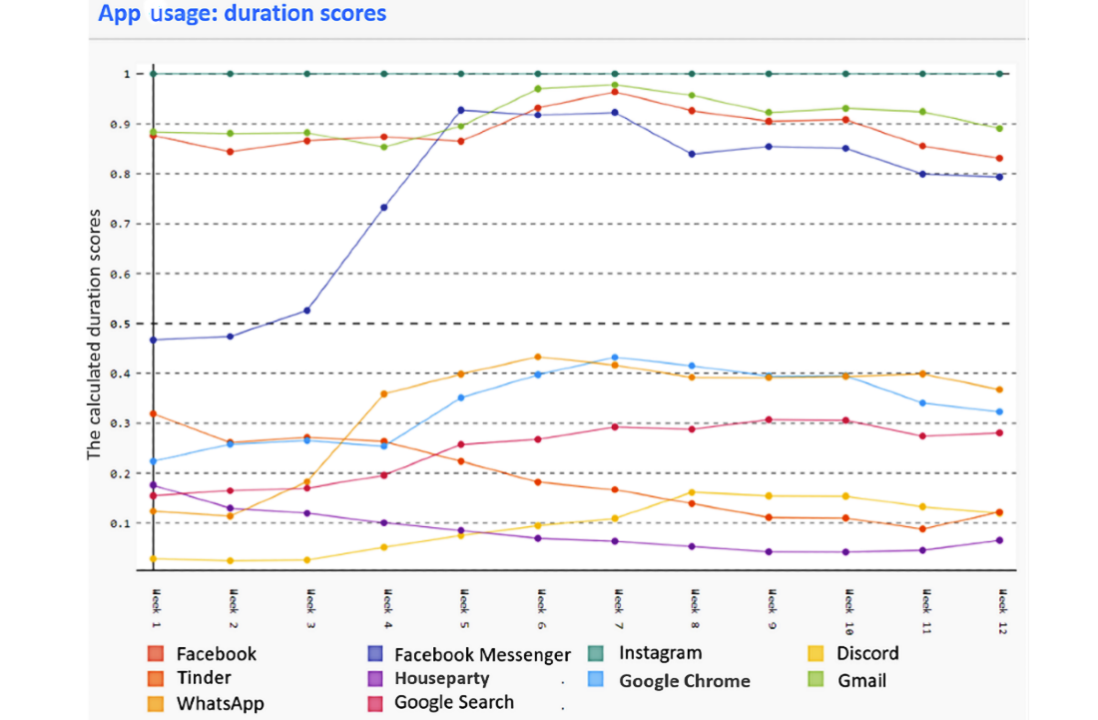
Normalized duration scores for a participant before and during the pandemic. The participant was enrolled in the first week of March and the lockdown started after the third week of data collection.

The results of our experiment show that the participants complied with COVID-19 policies. Participants managed to stay at home and adapt to the requested changes. However, to stay connected, the participant data show corresponding changes in app usage. The usage of social media apps, phone calls, and video conferences increased for most participants compared to the period before the lockdown. Figure 5 shows the app usage of a participant before and during the pandemic. Instagram was used the longest at 19.50 hours of usage, whereas the time spent on the Houseparty app was 9.27 hours. Values were normalized to easily observe the trend and be consistent with observations from other sources. The lockdown started during week 3. Consequently, the usage of apps, such as Facebook Messenger, WhatsApp, and Discord, has increased.

In contrast, 2 participants presented a decline in phone usage during the lockdown. When interviewed, the participants indicated that they started to use their personal computers and smart televisions more to accomplish the same tasks they previously did with smartphones.

## Discussion

### Principal Findings

The reported results show that actionable information can be derived from digital phenotyping. The information derived from understanding participants’ compliance, as well as the behavioral impact, can be used in personalized behavioral interventions. Behavioral nudges are used as an effective approach to promote behavioral changes. The NHS in the United Kingdom employs behavioral principles, such as reducing the cognitive load, to communicate nudges. We use actual text messages delivered by the NHS during the pandemic to demonstrate the potential benefit of personalization based on digital phenotyping. We show how a personalized understanding can be leveraged for more traction nudges and just-in-time intervention. The Behavioural Insights (BI) team [23] and the NHS have collaborated to nudge approximately 2 million people through text messages. The recipients of these nudges include people at the highest risk of developing critical complications should they contract the disease. The BI team employ the following behavioral principles to produce the content of a nudge (ie, the delivered text message).

1. Selection of the appropriate communication channel: since smartphone apps introduce multiple communication channels (eg, SMS, WhatsApp, and Messenger), personal preferences vary. The NHS and BI team have selected SMS as their preferred method on the basis of a study that shows that 85% of 600 participants do not mind receiving text messages on their personal devices from the NHS [24].
2. Signifying the key points: owing to the limitation of text messages, the NHS and BI team have to summarize extended guidelines into short messages. Accordingly, they designed messages such that the key ideas are prioritized.
3. Minimization of confusion and the cognitive load: the key ideas should be delivered in a language that is understandable by laypeople. Additionally, the messages should be clear to avoid confusion and misunderstanding that may quickly spread and negatively impact people.
4. Drawing on scientific behavioral findings: insights derived from behavioral and psychological studies are used to design nudges. For instance, it has been suggested that providing the rationale can help manage people’s mental health when quarantined. Accordingly, the NHS and BI team comply with that when designing nudges.

These behavioral principles are population-based, which has been reflected on the content of the nudge. M1, M2, and M3 (Table 2) are examples of 3 nudges that are delivered in accordance with these principles. We hypothesize that digital phenotyping can better improve the content and delivery of these nudges through personalization. For instance, the predicate of M1 can be tailored in accordance with the participant’s status as follows. We can predict whether or not a person lives alone from the digital phenotypes. Accordingly, 2 versions of the message can be prepared to deliver a personalized nudge. Versions can be tailored on the basis of the predicted status, age, or other demographics predictable through digital phenotyping.

**Table 2:** Text messages used by the National Health Service of the United Kingdom for nudging and our proposed personalization.

Code	Goal	Text message by the National Health Service of the United Kingdom	Personalization suggestions
M1	Nudge to establish social responsibility and stay connected	“If you live alone, text a friend or a family member to let them know you are following advice to stay at home until it is safer to mix with others. Plan to chat to someone over the phone at least once a day.”	If a participant chats regularly or lives with others, do not send the message and prevent overmessaging.
M2	Nudge to maintain a normal routine and ease anxiety	“Try to stick as closely as you can to your typical daily routine.”	If a participant frequents the cinemas, send the following message: “Watch a movie and try to stick as closely as you can to your typical daily routine.”
M3	Nudge to preserve mental health	“Are there things you enjoy doing at home that you usually don’t have time for?”	If a participant reports home activity, do not send the message and prevent overmessaging.

Digital phenotypes can also improve M2. For instance, an individual used to go to the cinema on Saturdays. Instead of delivering a general nudge about adhering to the typical routine, we can nudge the participant to watch a movie every Saturday during the pandemic. Thus, the typical routine can be embraced, and the delivery of the nudge can be contextualized (ie, just-in-time intervention). Adhering to typical routines can improve the mental health of individuals and reduce the negative impact of COVID-19 policies.

The information derived from digital phenotyping can also be used to prevent overmessaging. M1 encourages participants to chat with others to stay connected. If the derived data show that a participant regularly chats with others, there is no need to send M1. We speculate that crafting messages on the basis of both data and behavioral principles as well as introducing fewer messages is expected to provide better results. However, actual field testing is required to scientifically measure the real effect of doing so.

Although our approach demonstrates a potential way of producing personalized nudges, it can be reflected in existing behavioral change frameworks such as the behavioral change wheel [25]. For instance, the framework of the behavioral change wheel identifies 3 main stages to the behavioral change: (1) understanding the behavior to be changed, (2) deciding on the intervention function, and (3) selecting the mode of delivery. We profile and understand the individuals’ behaviors through digital phenotyping. Incentivization and persuasion are intervention functions that shape nudging [18]. Communication as a delivery mode is then used to deliver text messages that nudge people to exhibit the desired behavior.

We are aware of the privacy concerns that may hinder the measurements and implementation of personalized nudges. However, apps can be designed in a way that allows people to partially share information in accordance with their needs. For instance, an individual may choose to share the location data only if diagnosed with COVID-19, to trace and limit the spread of the disease to others. Another individual may choose to share his/her data to receive personalized nudges that help him/her adhere to the daily routine (M2). Nevertheless, in these cases and others, personal behaviors are privately phenotyped, and it is up to the person whether or not to share the collected data. Alternatively, messages can be packaged with the app and delivered to participants on the basis of the outcome of a decision tree.

Stay-at-home, social distancing, and other policies are primarily behavioral measures aimed at changing individuals’ behaviors to ensure that the risk of contracting the disease is reduced. From this standpoint, behavioral change frameworks (eg, nudging and the behavioral change wheel) can be relied upon to support the implementation of these behavioral policies. The use of digital phenotyping in activating these frameworks provides an opportunity to personalize the delivery of these policies on the basis of each individual’s data. Individuals, institutions, and governments can benefit from such personalization in containing the spread of the virus. Governments may choose to develop apps that have behavioral policies implemented as built-in messages. The delivery of these messages is designed to adapt in accordance with the exhibited behaviors. Individuals who stayed at home (according to digital phenotyping) will not receive messages encouraging them to do so. This decision and others related to message delivery are made locally, on the individual’s phone, without compromising his/her privacy. However, individuals who test positive can help governments reduce the potential impacts on others by voluntarily sharing their latest mobility behaviors.

Besides generating personalized nudges, digital phenotyping shows its capability to observe people’s behavior on an individual level. In the contest of the COVID-19 pandemic, digital phenotyping has great potential for various implementations. Some of the COVID-19 tracking apps such as TraceTogether in Singapore and COVIDSafe in Australia have used Bluetooth technology embedded in smartphones as their primary contact tracing tool [26]. People are encouraged to install these apps so they can know if they have been in close contact with individuals who have tested positive for COVID-19. Institutions such as universities can implement digital phenotyping as innovative methods to study the traditional physiological or societal questions, since no face-to-face settlement is needed. Care facilities could also have digital phenotyping apps installed on their clients’ smartphones, such that their issues can be noted without face-to-face reporting. Moreover, the large amount of personal and longitudinal digital phenotyping data could provide policymakers with a deeper understanding of the impact of COVID-19 on a sample of the population. This would shed light on how people actually react to these policies, rather than only determining the infection rate.

### Conclusions

This study shows how digital phenotyping can be of value in understanding people’s behavior during a pandemic. Behavioral features extracted from digital phenotypes represent the cornerstone that facilitates the personalization of and compliance with behavioral policies. We presented examples of using Bluetooth, GPS, and app usage data to analyze behavioral responses to COVID-19 policies. Additional sources can be further investigated, such as accelerometers and their role in understanding if people pause more to maintain safe distance.

To encourage the large-scale adaptation of digital phenotyping, governments can emphasize the potential benefits of public health and of maintaining mental health. To preserve privacy, an individual’s data are stored locally, and he/she can make the ultimate decision on what to share and to whom the access is granted.

A rule-based messaging implementation can be used to deliver nudges on the basis of the analysis of digital phenotyping. In future studies, we intend to examine the impact of these suggested messages on a sample of the population to measure the impact of preventing overmessaging. Conducting a real-world experiment would also enable us to assess whether having more tailored messages would yield the expected benefits.

## Abbreviations

BI: Behavioural Insights
GPS: global positioning system
NHS: National Health Service
